# Time-trend of hospitalizations for anogenital warts in Veneto region in the HPV vaccination era: a cross sectional study (2007–2018)

**DOI:** 10.1186/s12879-020-05591-6

**Published:** 2020-11-18

**Authors:** S. Cocchio, G. M. Prandi, P. Furlan, C. Bertoncello, M. Fonzo, M. Saia, T. Baldovin, V. Baldo

**Affiliations:** 1 Department of Cardiac Thoracic Vascular Sciences and Public Health, University of Padua, Padua, Italy; 2 Department for Woman and Child Health, University of Padua, Padua, Italy; 3 “Azienda Zero” of Veneto region, Padua, Italy

**Keywords:** HPV, Hospitalization, Genital warts, Vaccination

## Abstract

**Background:**

Human papillomavirus (HPV) is a common sexually transmitted pathogen and the cause of several cancers and of anogenital warts. With this study, we estimated the trend of hospitalizations for anogenital warts (AGWs) in the Veneto region (Italy) from 2007 to 2018.

**Methods:**

The analysis included all the hospital discharge records of public and accredited private hospitals occurred in Veneto residents in the timespan 2007–2018. The ICD9-CM code 078.11 considered were those associated with condyloma acuminatum and those associated with surgical interventions for vulval/vaginal warts, penile warts anal warts. Annual total and sex- and age-specific hospitalization rates and trends were calculated and correlated with the different HPV vaccine coverage over the study period.

**Results:**

We observed an overall reduction of hospitalization rates for AGWs: from 15.0 hospitalizations every 100,000 Veneto residents in years 2007–08 to 10.9 hospitalizations every 100,000 Veneto residents in year 2017–18 (− 37.4%; *p* < 0.05). Reduction has been caused by a drop in hospitalizations in females - from a rate of 20.4/100,000 in 2007–2008 to a rate of 10.8/100,000 in 2017–18 (AAPC: -7.1; 95%CI: − 10.6;-3.4); while in males, we observed a slight - but not statistically significant - increase in hospitalization rates.

**Conclusion:**

The marked decline in hospitalization rates for AGWs in Veneto Region is probably attributable to the high coverage rates of HPV vaccination programs implemented since 2008.

## Background

Human papillomavirus (HPV) is one of the most frequently sexually transmitted viruses in the world. HPV infection plays a critical role in the pathogenesis of many forms of cancer (cervical, anal, vulvar, vaginal, penile, and head-neck cancers) as well as in the development of common dermatologic and sexually transmitted diseases, first of all condylomata acuminata or anogenital warts (AGWs) [1]. Anogenital warts cause a deep impact on the affected patient’s quality of life and their treatment requires, in some cases, hospitalization [2]. The first two vaccines (tetra-valent and bi-valent) designed to prevent papillomavirus-related diseases were licensed by European Medicines Agency (EMA) in 2006 and 2007 respectively, followed by the nine-valent vaccine licensed by EMA in 2015 [3]. In 2007, the Italian Minister of Health through its technical committee, Consiglio Superiore di Sanità, unanimously provided a positive opinion on HPV vaccination [4]. The committee indicated the 12-year old female cohort as priority with the possible extension to the 25/26-year old female cohort [4]. From 2008, Italian Regions implemented the HPV vaccination program in the 12-year old female cohort and some of them extended free catch-up vaccination to one or more older cohorts (16- year old, 18-year old, and 25-year old cohorts) [5].

Veneto Region started to provide HPV vaccination with the quadri-valent vaccine for free and with an active-call system to females of 12 years of age in 2008 [6]; from 2014 the vaccination has been extended to males [7] and from 2017 the gender-neutral vaccination has been practiced with the nine-valent vaccine [8].

Hospital Discharge Records (HDRs) are a useful tool for assessing the reporting of cases of those AGWs with such a severe presentation to warrant admission to hospital [9]. In developed countries, the percentage of patients with AGWs hospitalized varies between circa 7% and circa 19 [10–12]. Patients undergo hospitalization only in the most severe and complex cases that require surgical excision of the AGWs [13].

The present study aims to analyze the temporal trend of genital warts-related hospitalizations and evaluating the impact of HPV vaccination on hospitalization rates in the Veneto Region between 2007 and 2018. In addition, given that AGWs’ reduction is an early indicator of the long-term effect of the vaccination [14], data may provide a glimpse about what can be expected about the clinical impact on vaccine-type HPV-related cancers.

## Methods

We conducted the analysis on data from January the 1st 2007 to December the 31st 2018 in Veneto Region. Published reports and HDRs of Veneto’s database were checked to include codes of the International Classification of Diseases, Ninth Revision, Clinical Modification (ICD-9-CM) related to all HPV-related diseases. Through HDRs, we collected demographic data (date of birth, gender, nationality, and place of residence) and clinical information (surgery ablative interventions in anogenital area, date of discharge). Data from HDRs were included in the analysis if the related ICD9-CM code 078.11: condyloma acuminatum was present in the primary (first-listed) diagnosis or in one of the secondary diagnosis. As for surgical interventions ICD9-CM codes, we included 70–71 and 58.3 (vulval/vaginal warts), 64 and 58.3 (penile warts) and 49 (anal warts) codes. HIV positivity was evaluated by researching the ICD9-CM 042 code in all diagnoses.

We considered repeated admissions those cases of 2 hospitalizations for AGWs in a 30 day-period, and in those events only the first hospital stay was considered in the analysis. Annual hospitalization rates for AGWs were estimated by dividing the annual number of AGWs hospitalizations by the population of Veneto residents in the designated year as per data from Veneto Regional Authority of Statistics office. We conducted analysis by using two-year intervals and four age-groups (17–20 years, 21–26 years; 27–46 years and older than 47 years of age at the time of the hospital admission). This stratification by age allowed us to analyze directly the trend of AGWs in age-groups for entire study period, as well as having a first hint of the effect of vaccination in the 17–20 years of age group.

The first birth cohort, 1996, of females vaccinated in 2008 reached 17 years of age in 2013 and we have been able to observe the group for a total of 6 years (4 years of data included in the 17–20 age group, and 2 years of data included in the 21–26 age group), the 1997 birth cohort has been observed for a total of 5 years (4 years of data included in the 17–20 age group, and 1 year of data included in the 21–26 age group) and so on up to 2002 birth cohort, the last cohort to have data included in the analysis.

### Statistical analysis

Data were analyzed by using Student’s t-test for continuous data and Pearson’s chi square test for categorical data. A *p* value < 0.05 was considered significant. The analyses were performed using the Statistical Package for the Social Sciences (SPSS 22.0; SPSS Inc., Chicago, IL, USA). Significant trends over the years considered were assessed as average annual percent changes (AAPC), a summary measure of the trend over a given fixed interval that is computed as a weighted average of the annual percent change (APC) emerging from the joint-point model, using weights equating to the length of the APC interval. If an AAPC lies entirely with a single joint-point segment, the AAPC is the same as the APC for that segment [15].

## Results

From 1 January 2007 to 31 December 2018, 6977 hospitalizations related to an AGWs diagnosis in one of the primary or secondary fields were registered, 104 of them have been excluded from the analysis being repeated admissions. Therefore, the analysis included data from 6873 subjects.

Among the 6873 patients whose data were included in the analysis, females were the 58.2% (4000) and males were the 41.8% (2873). The mean age was 36.5 ± 12.8 years, the female’s mean age being 34.7 ± 12.3 and male’s mean age being 39.0 ± 13.3. Overall, HIV positivity was recorded in 0.4% of sample (Table 1).

**Table 1:** Characteristics of the 6873 patients

	Males (***n*** = 2873)		Females (***n*** = 4000)		Total (***n*** = 6873)	
	n	(%)	n	(%)	n	(%)
**Period**						
**2007–2008**	460	(16.0)	924	(23.1)	1384	(20.1)
**2009–2010**	477	(16.6)	933	(23.3)	1410	(20.5)
**2011–2012**	475	(16.5)	627	(15.7)	1102	(16.0)
**2013–2014**	436	(15.2)	528	(13.2)	964	(14.0)
**2015–2016**	498	(17.3)	525	(13.1)	1023	(14.9)
**2017–2018**	527	(18.3)	463	(11.6)	990	(14.4)
**Age group**						
**0–16**	8	(0.3)	27	(0.7)	35	(0.5)
**17–20**	61	(2.1)	249	(6.2)	310	(4.5)
**21–26**	456	(15.9)	975	(24.4)	1431	(20.8)
**27–46**	1607	(55.9)	2116	(52.9)	3723	(54.2)
**47+**	741	(25.8)	633	(15.8)	1374	(20.0)
**HIV positive**	16	(0.6)	9	(0.2)	25	(0.4)
**Nationality**						
**Italian**	2702	(94.0)	3550	(88.8)	6252	(91.0)
**Foreign**	171	(6.0)	450	(11.3)	621	(9.0)

In the time period of the analysis, we observed an overall reduction of hospitalization rates for AGWs: from 15.0 hospitalizations every 100,000 Veneto residents in years 2007–08 to 10.9 hospitalizations every 100,000 Veneto residents in year 2017–18 (− 37.4%; *p* < 0.05). In females, the overall reduction has been of 88.9%, with a rate of 20.4 per 100,000 in 2007–2008 to a rate of 10.8 per 100,000 in 2017–18 (AAPC: -7.1; 95%CI: − 10.6;-3.4); in males, on the contrary, we detected only an insignificant increase (AAPC: 0.9; 95%CI: − 1.1; 2.9).

**Fig. 1:**
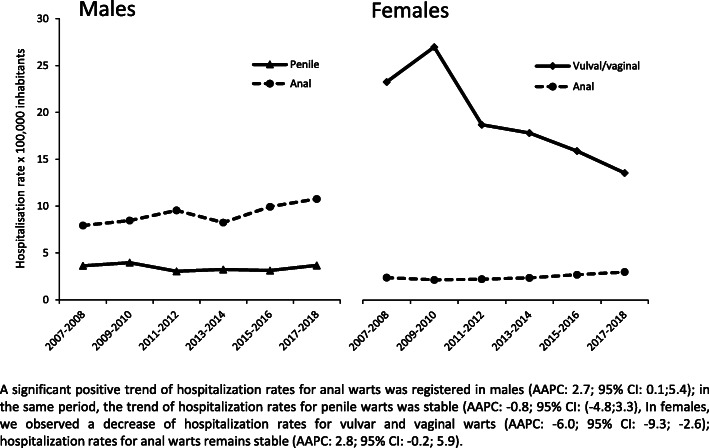
Male and female anogenital wards trend (per 100,000 inhabitants) in the Veneto Region

The most frequent presentation of anogenital warts in males was in the anal anatomical site, being the presentation in 2108 out of 2873 of cases (70.2%); penile warts accounted for a total of 397 cases (13.8%) and in 368 cases the anatomical site was not provided. In the observation period, data showed a positive trend for the total number of hospitalizations due to AGWs in the male population, a trend driven by the rise of anal condyloma. The hospitalization rate of anal warts in males showed a significant increase from a 7.9 per 100,000 in 2007–08 to 10.8 per 100,000 in 2017–18 (AAPC: 2.7; 95% CI: 0.1;5.4). In the same period, the trend of hospitalization rates for penile warts was stable (AAPC: -0.8; 95%CI: − 4.8; 3.3) (Fig. 1).

**Fig. 2:**
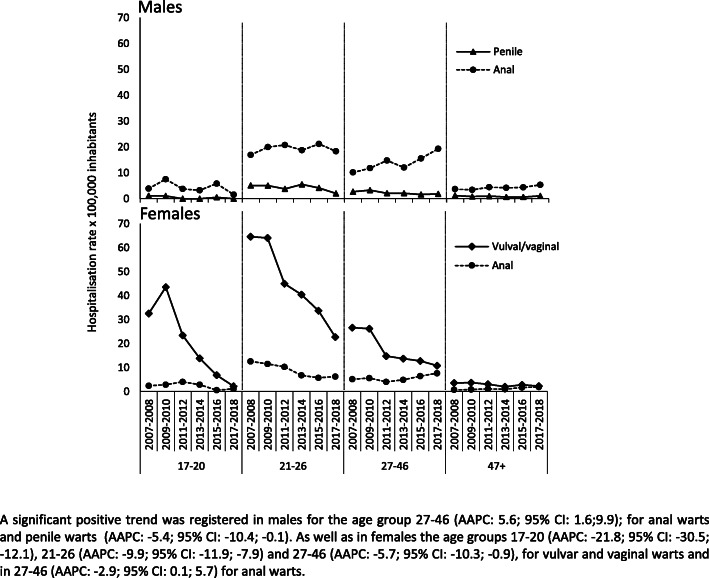
Trend of hospitalization for anogenital warts rates in different age groups according to gender

Looking at the trend of hospitalizations for the different age groups, we observed a statistically significant increase of hospitalization rates only in the age group 27–46. In the 27–46 group, in fact, the rate went from 16.2 per 100,000 in the time period 2007–2008 to 23.1 per 100,000 in the time period 2017–2018 (AAPC: 2.9; 95%CI: 0.1;5.7), such a rise was driven by the increase in hospitalizations for anal warts that went from 10.2 per 100,000 in 2007–2008 to 19.3 per100,000 in 2017–2018. All other age groups did not show a statistically significant modification of hospitalization rates, however, we observed, starting from 2015 to 2016, a slight decrease in hospitalization rates in the age groups 17–20 and 21–26. In details, in the 21–26 age group, hospitalization rate for anal and penile warts went from 21.2 per 100,000 in 2015–16 to 18.4 per 100.000 in 2017–2018 and from 4.2 per 100,000 in 2015–2016 to 2.1 per 100,00 in 2017–2018, respectively; in the 17–20 age group, anal warts hospitalization rate decreased from 5.8 per 100,000 in 2015–2016 to 1.6 per 100,000 in 2017–2018 and we observed no variation for penile warts (Fig. 2).

The most frequent presentation of warts in females was in the genital anatomical site, being the presentation in 2899 out of 4000 of cases (72.5%); anal warts accounted for a total of 821 cases (13.8%) and in 280 cases the anatomical site was not provided.

In the observation period, data showed a clear negative trend for the total number of hospitalizations due to AGWs in the female population, a tendency driven mostly by the decrease of vulvar and vaginal warts (AAPC: -6.0; 95%CI: − 9.3; − 2.6). Indeed, the overall hospitalization rate of anal warts in females, showed not significant trend (AAPC: 2.8; 95% CI: − 0.2;5.9) (Fig. 1).

Investigating the trend of hospitalizations for the different age groups, a statistically significant decrease of hospitalizations rates emerged in all age groups except for the ≥47-year old subjects, a group that, in any case, showed low but fluctuant values (Fig. 2). The most dramatic decrease occurred in the 17–20 age group where hospitalization rates dropped from values of 38.0 per 100,000 in 2007–2008 and of 47.6 per 100,000 in 2009–2010 to a minimum of 3.9 per 100,000 in 2017–2018 (AAPC: -21.8; 95%CI: − 30.5;-12.1), the drop driven by the decrease in hospitalizations for vulvar/vaginal warts (AAPC: -24.3; 95%CI: − 34.4;-12.7). The 21–26 age group, too, showed a noteworthy decrease in AGWs hospitalization rates going from a value of 84.1 per 100,000 in 2007–2008 to a rate of 30.4 in 2017–2018 (AAPC: -9.9^; 95%CI: − 11.9; − 7.9). In this case, we observed reductions both in the anal warts (AAPC: -8.2; 95%CI: − 12.1; − 4.2) and genital warts (AAPC: -9.8; 95%CI: − 12.9; − 6.7). Finally, in the 27–46 age group, rates went from 34.1 per 100,000 of 2007–2008 to 20.0 per 100,000 in 2017–2018 (AAPC: -5.7^; 95%CI: − 10.3; − 0.9). Considering the anatomical site, the 27–46 age group showed a reduction only in the genital anatomical site (AAPC: -9.2; 95%CI: − 13.5; − 4.7) while we observed a minor increase in anal warts hospitalization rates. (Fig. 2).

## Discussion

In the time period from January 2007 to December 2018, we observed an overall decrease of hospitalizations for genital warts in both sexes, not counterbalanced by the global increase of hospitalizations for anal warts. The aggregated rate of hospitalizations for both sexes was 14.4 per 100,000 in 2007 and 9.6 per 100,000 in 2018, a modification compatible with an average annual percent change of − 4.0% (95% CI: − 5.6%; − 2.4%). The rate in male population stayed almost stable across the 12 years, with a value of 8.8 per 100,000 in 2007 and a value of 10.7 per 100,000 in 2018 (AAPC: 1.1; 95% CI: − 0.5; 2.7). Anyway, a significant increase was recorded in males for age group 27–46 (AAPC: 5.6; 95% CI: 1.6;9.9). As evidenced by literature data, anal warts HPV related is highly prevalent in men who have sex with men [16].

In our analysis, many factors may have played a role in the positive trend of genital warts observed in men aged 27–46. A 2015 cross-sectional survey conducted on 11,096 students aged ≥18 years highlighted differences in sexual behaviors and health habits among between genders. Among those differences, the most notable are a lower frequency of exclusive relationship in males vs females (40.0% vs 57.8%, *p* < 0.001), an higher mean number of sex partners in males vs females in the last 24 months (1.9 ± 2.3 vs 1.3 ± 1.4, *p* < 0.001), and lower HPV-vaccination rates in males vs females (1.3% vs 36.8%) [17].

A previous study of Cocchio et al., 2017 [18], of which the present research constitutes a continuation, already highlighted a decline in the rate of AGWs-related hospital admissions in a population with a good HPV vaccination coverage. Cocchio and colleagues described a significant reduction, starting from 2013, in hospitalizations for vulvar/vaginal warts in patients 12–20 years old.

The data emerging from the present study helped to further outline the above-mentioned trend for a longer time period also in the narrower group 17–20, as well as in the older group of 21–26 years of age. Both those groups benefitted from the high vaccination coverage rates (up to 70%) reached in females [19].

In males, data did not show similarly significant decreases in hospitalizations neither for genital or anal warts for almost the entire period. The last 2 years of analysis, however, seem to provide in the younger groups (17–20 and 21–26) a first hint of a beginning of decrease in hospitalization rates parallel to the one observed in females. This observation may find two not mutually exclusive explanations: the start of the gender-neutral vaccination in 2015 and the herd effect, as described in other countries [11].

The significant reduction of hospitalization rates for anogenital warts in females and the slower decrease shown in males are consistent with the conclusions of a recent systematic review and meta-analysis [20]. Drolet and colleagues described a picture common to those countries that implemented the quadrivalent vaccination in females: in the first 4 following years a significant decrease in anogenital warts occurred in females aged 15–29 years while non-significant but substantial decreases occurred in males aged 15–19 years [20].

A matter of interest for future studies will be to observe how the modifications in hospitalization rates will be affected by the gender-neutral vaccination, introduced since 2014 in Veneto Region and that allowed to reach coverage rates that in male 2004 birth-cohort reached the value of 60% [19].

The present study shows two main strengths: in the first place, it includes data from a twelve-year timespan with eleven years of data collection in vaccinated cohorts and three cohorts followed for at least 4 years (birth cohort of 1997 followed for a total of 6 years, birth cohort od 1998 followed for 5 years and birth cohort of 1999 followed for 4 years). The second strength is the chance to obtain data in a region with high female coverage rates and therefore to observe changes that require a short period to intervene. On the other hand, we believe the study may suffer from three limitations: first, hospital records may not automatically correspond to the total number of HPV cases identified for inaccuracy in coding in the hospital records or for unregistered records at all. By checking our data for AGWs-related surgical codes we should have minimized the effect of such selection bias. Second, diagnoses did not undergo a double-check because of the lack of cytological and/or microbiological analysis data. Finally, by using HDRs we were not able to speculate about differences, if any, in sexual behaviors in the different groups studied.

AGWs that undergo treatment in hospital represent a part of overall AGW burden and that is those with such a severe presentation to warrant admission to hospital. Therefore, data presented in the study cannot be intended as informative of trends in the broader community setting. However, in the years 2014–2018 numbers of AGWs observed by the sentinel Italian surveillance of sexually transmitted infections remained stable [21].

## Conclusions

The overall decrease of hospitalization rates for AGWs indicates of a positive trend after the introduction of the HPV vaccination. As expected, the reduction has been more pronounced in those Veneto residents who were offered free vaccination, i.e. younger females. However, we observed a first indication of benefit in males of the same age, that may indicate a possible herd immunity effect. Further studies collecting data in the next years will be necessary to confirm the reduction trend in females and to demonstrate a similar tendency in males.

## Data Availability

All relevant data are within the paper. Requests for additional information should be addressed to the corresponding author and data may be provided on reasonable request.

## Abbreviations

HPV: Human papillomavirus
AGWs: Anogenital warts
ICD9-CM: International Classification of Diseases, Ninth Revision, Clinical Modification
EMA: European Medicines Agency
HDRs: Hospital Discharge Records
AAPC: Average annual percent changes
APC: Annual percent change

## References

[1] Brianti P, De Flammineis E, Mercuri SR (2017). Review of HPV-related diseases and cancers. New Microbiol.

[2] Grennan D (2019). Genital warts. JAMA..

[3] Lopalco PL (2016). Spotlight on the 9-valent HPV vaccine. Drug Des Devel Ther.

[4] Consiglio Superiore di Sanità, Sessione XLVI, Sessioni congiunte II e III, seduta dell’ 11 gennaio 2007, http://www.salute.gov.it/imgs/C_17_pubblicazioni_600_allegato.pdf. Accessed 08 Oct 2020.

[5] Cappelli MG, Fortunato F, Tafuri S, Boccalini S, Bonanni P, Prato R, Martinelli D (2018). Cervical cancer prevention: an Italian scenario between organised screening and human papillomaviruses vaccination. Eur J Cancer Care (Engl).

[6] Giambi C, Declich S, Finarelli AS, Pascucci MG, Salmaso S. e il Gruppo di Sanità Pubblica del Coordinamento Interregionale della Prevenzione. Strategie vaccinali nazionali e regionali per la vaccinazione anti-HPV e primi dati di copertura vaccinale: a che punto siamo. Epicentro. https://www.epicentro.iss.it/ben/2010/novembre/2. Accessed 08 Oct 2020.

[7] Favaretti C, de Waure C, Poscia A, Sacchini D, De Vincenzo R, Bechini A, Boccalini S, Bonanni P, Zanella B, Conversano M, Battista T, Giorgino A, Russo C, Ferro A, Mennini FS, Marcellusi A, Baio G, Nardi S, Squillace A (2017). Il vaccino anti-HPV 9-valente: report di Health. Technol Assess (HTA) QIJPH.

[8] DECRETO N. 89 del 17.05.2017, https://bandi.regione.veneto.it/Public/Download?idAllegato=5685. Accessed 08 Oct 2020.

[9] Boehmer TK, Patnaik JL, Burnite SJ, Ghosh TS, Gershman K, Vogt RL (2011). Use of hospital discharge data to evaluate notifiable disease reporting to Colorado's electronic disease reporting system. Public Health Rep.

[10] Marra F, Ogilvie G, Colley L, Kliewer E, Marra CA (2009). Epidemiology and costs associated with genital warts in Canada. Sex Transm Infect.

[11] Hillemanns P, Breugelmans JG, Gieseking F, Bénard S, Lamure E, Littlewood KJ, Petry KU (2008). Estimation of the incidence of genital warts and the cost of illness in Germany: a cross-sectional study. BMC Infect Dis.

[12] Ali H, Guy RJ, Wand H, Read TR, Regan DG, Grulich AE, Fairley CK, Donovan B (2013). Decline in in-patient treatments of genital warts among young Australians following the national HPV vaccination program. BMC Infect Dis.

[13] Scheinfeld N, Lehman DS (2006). An evidence-based review of medical and surgical treatments of genital warts. Dermatol Online J.

[14] Mariani L, Vici P, Suligoi B, Checcucci-Lisi G, Drury R (2015). Early direct and indirect impact of quadrivalent HPV (4HPV) vaccine on genital warts: a systematic review. Adv Ther.

[15] Kim HJ, Fay MP, Feuer EJ, Midthune DN (2000). Permutation tests for joinpoint regression with applications to cancer rates. Stat Med.

[16] Jin F, Prestage G, Kippax SC, Pell CM, Donovan B (2007). T DJ, Kaldor J, Grulich AE. Risk factors for genital and anal warts in a prospective cohort of HIV-negative homosexual men: the HIM study. Sex Transm Dis.

[17] Cocchio S, Bertoncello C, Baldovin T, Buja A, Majori S, Baldo V (2018). Self-reported genital warts among sexually active university students: a cross-sectional study. BMC Infect Dis.

[18] Cocchio S, Baldovin T, Bertoncello C, Buja A, Furlan P, Saia M, Baldo V (2017). Decline in hospitalization for genital warts in the Veneto region after an HPV vaccination program: an observational study. BMC Infect Dis.

[19] Coperture vaccinali al 31/12/2017 per HPV (Aggiornamento 2 luglio 2018), http://www.salute.gov.it/imgs/C_17_tavole_27_allegati_iitemAllegati_0_fileAllegati_itemFile_1_file.pdf. Accessed 08 Oct 2020.

[20] Drolet M, Bénard É, Pérez N, Brisson M (2019). HPV vaccination impact study group. Population-level impact and herd effects following the introduction of human papillomavirus vaccination programmes: updated systematic review and meta-analysis. Lancet..

[21] Salfa MC, Ferri M, Suligoi B e la Rete Sentinella dei Centri clinici e dei Laboratori di microbiologia clinica per le Infezioni Sessualmente Trasmesse (2020). Le Infezioni Sessualmente Trasmesse: aggiornamento dei dati dei due Sistemi di sorveglianza sentinella attivi in Italia al 31 dicembre 2018. Not Ist Sup Sanit.

